# A scoping review of utilization of the verbal fluency task in Chinese and Japanese clinical settings with near-infrared spectroscopy

**DOI:** 10.3389/fpsyt.2024.1282546

**Published:** 2024-03-08

**Authors:** Yufei Ren, Gang Cui, Kun Feng, Xiaoqian Zhang, Chenchao Yu, Pozi Liu

**Affiliations:** ^1^ Department of Foreign Languages and Literatures, Tsinghua University, Beijing, China; ^2^ Department of Psychiatry, Yuquan Hospital, Tsinghua University, Beijing, China; ^3^ Independent Practitioner, Beijing, China; ^4^ School of Clinical Medicine, Tsinghua University, Beijing, China

**Keywords:** verbal fluency task, prefrontal lobe, functional near-infrared spectroscopy, psychiatric disorders, brain and language

## Abstract

This review targets the application of the Verbal Fluency Task (VFT) in conjunction with functional near-infrared spectroscopy (fNIRS) for diagnosing psychiatric disorders, specifically in the contexts of China and Japan. These two countries are at the forefront of integrating fNIRS with VFT in clinical psychiatry, often employing this combination as a complementary tool alongside traditional psychiatric examinations. Our study aims to synthesize research findings on the hemodynamic responses elicited by VFT task in clinical settings of the two countries, analyzing variations in task design (phonological versus semantic), stimulus modality (auditory versus visual), and the impact of language typology. The focus on China and Japan is crucial, as it provides insights into the unique applications and adaptations of VFT in these linguistically and culturally distinct environments. By exploring these specific cases, our review underscores the importance of tailoring VFT to fit the linguistic and cultural context, thereby enhancing its validity and utility in cross-cultural psychiatric assessments.

## 1 Introduction

The Verbal Fluency Task (VFT) is one of the most frequently used neuropsychological tests (1), in which participants are asked to generate as many words as possible in a given time, starting with a given letter or on the basis of a semantic category (2). It is frequently used in non-clinical groups, measuring the efficiency of word generation and assesses the effectiveness of language use in information transfer, thereby examining executive functions, such as working memory, response inhibition, and cognitive flexibility (2–6). For its neurological underpinnings, many studies have reported VFT as a sensitive indicator of deficits in prefrontal and temporal regions (7–9). Moreover, VFT is extensively employed in studies assessing cognitive deficits in patients with mental disorders (10–15).

Several advanced functional imaging techniques have been used to uncover the neurological underpinnings of VFT, among which functional near-infrared spectroscopy (fNIRS) has been leveraged in healthcare and medical research. fNIRS constitutes an innovative functional neuroimaging method that enables non-invasive tracking of changes in concentrations of oxygenated hemoglobin [oxy-Hb] and deoxygenated hemoglobin [deoxy-Hb] within the microvasculature of brain tissue during cognitive activity. This is achieved through real-time monitoring of neural activity proximal to the brain’s surface (16, 17). fNIRS, an emerging neuroimaging tool, offers distinct advantages in brain research and clinical applications. Its various modes—continuous wave (CW), frequency domain (FD), and time domain(TD)—provide diverse insights into cerebral hemodynamics (18). CW-fNIRS, the most common due to its simplicity and cost-effectiveness, uses light with constant frequency and amplitude, measuring relative changes in hemoglobin oxygenation but not providing absolute measurements due to the unknown photon path-length (19). FD-fNIRS, employing amplitude-modulated NIR laser sources, offers more complexity and cost but can measure absolute concentrations of hemoglobin by assessing the back-scattered signal’s amplitude and phase shift (20). TD-fNIRS, the most technically complicated and expensive, uses short NIR pulses to directly observe photon path-length, providing the most accurate baseline hemoglobin concentration and oxygenation values (21).

Functional Magnetic Resonance Imaging (fMRI) and Electroencephalography (EEG) are also pivotal in the realm of neuroimaging, offering insights into the brain’s functional and structural aspects. fMRI, a non-invasive modality, excels in mapping brain activity by tracking changes in blood oxygen levels, termed Blood Oxygen Level Dependent (BOLD) contrast. This change is a marker of neural activity, with more active brain regions consuming increased oxygen, a phenomenon captured by fMRI (22). Conversely, EEG specializes in recording the brain’s electrical activity. It measures voltage fluctuations caused by ionic current flows in brain neurons, crucial for studying temporal aspects of brain function and diagnosing neurological conditions like epilepsy (23).

Integrating the discussion of functional Near-Infrared Spectroscopy (fNIRS) highlights the evolution in neuroimaging. fNIRS, compared to fMRI, offers superior temporal resolution, allowing for more detailed tracking of neural dynamics (16). It also outperforms EEG in spatial resolution, pinpointing brain activity locations with greater accuracy (17). The low operational cost, silent operation, and non-restrictive nature of fNIRS make it particularly user-friendly and suitable for diverse demographics, including children (24). Its potential for multimodal imaging further enhances its research utility (25). The broad applicability of fNIRS, unhampered by age or other common research exclusions, underscores its significant role in inclusive neuroscientific studies (13, 26). Hemodynamic assessment utilizing fNIRS during cognitive tasks potentially serve as a promising biomarkers in personalized psychiatric practice (27). Differences in fluctuations of [oxy-Hb] and [deoxy-Hb] in various brain regions may characterize distinct types of psychiatric disorders (28–34).

Over a decade, fNIRS has increasingly been employed to enhance clinical interviews and mental state evaluations in diagnosing and monitoring psychiatric conditions (30, 35). Notably, Japan and China stand out as leading countries in integrating VFT with fNIRS in actual clinical settings, supplementing the assessments of experienced psychiatrists and extending its use beyond academic research. In both nations, large-scale patient populations (exceeding thousands) routinely undergo VFT-fNIRS examinations as part of their clinical diagnosis, as documented by Takizawa et al. (10) and Li & Liu (36). The majority of fNIRS research and application in psychiatry is attributed to Japanese scholars, with about two-thirds of the foundational articles on this topic originating from Japan (37). A comprehensive review by Ho et al. (35) indicates that most studies on the diagnostic and predictive capabilities of fNIRS for Major Depressive Disorders have been conducted in Japan. More recently, China has adopted fNIRS in psychiatric clinical diagnosis, particularly in regions with a large Chinese-speaking population. Analysis of research papers reveals that Japan leads in studies using fNIRS to distinguish depressed patients from healthy controls, followed by China and Germany (35). However, China faces unique challenges in clinical diagnosis, particularly in adapting VFT for its demographic due to the lack of a phonemic fluency test specifically designed for the Chinese language.

Unlike alphabetical languages such as English, where words begin with letters, Chinese uses a logographic system where each character represents a word and is often associated with a pictorial symbol. This fundamental difference in linguistic structure, exemplified by the stroke-based composition of Chinese characters (38), raises questions about how language specifics might influence neurological activations as measured by fNIRS.

Given the diversity of languages and their unique characteristics, as seen in adaptations of VFT for Portuguese (39), Dutch (40), Arabic (41), and Thai (42), it becomes imperative to examine the design and implementation of VFT in fNIRS applications across different languages.

Variations in VFT designs may pose diagnostic challenges, underscoring the need for uniform paradigms, protocols, and analytical methodologies to guarantee the reproducibility and validity of outcomes. The selection of VFT significantly influences results due to varying word frequencies and letter complexities across languages, as noted by Strauss et al. (43). Establishing standardized VFT approaches aligns with the aims of a scoping review, as described by Tricco et al. (44), emphasizing comprehensive evaluation and synthesis of research in this field.

This paper aims to examine how VFT patterns are differentially implemented in clinical settings for psychiatric diagnosis in China and Japan. This comparative study will contribute to understanding the role of neurological activation in language processing and inform the future development and application of fNIRS in psychiatric diagnosis and management. Additionally, establishing an international community of NIRS researchers could enhance the utilization and expertise of fNIRS in this challenging area.

## 2 Methods

This review has been designed in accordance with the latest guidelines from the Joanna Briggs Institute for conducting scoping reviews (45). To ensure a standardized and transparent reporting process, we have adopted the Preferred Reporting Items for Systematic reviews and Meta-Analyses extension for Scoping Reviews (PRISMA-ScR) guidelines, as proposed by 46. This approach guarantees a rigorous and comprehensive exploration of the available literature, adhering to the highest standards in systematic review methodology.

### 2.1 Eligibility criteria

In terms of eligibility for this scoping review, we placed no restrictions on the publication date. However, we only considered studies that were published in English or had an English translation available. In regard to the sample, only studies involving human participants were selected. With regard to the study design, only original studies were considered, and review articles were consequently excluded. Case reports or studies with a sample size of less than five were not considered for this review. In relation to the measures of prefrontal activation, we solely considered cortical hemodynamic measures obtained through the NIRS technique. We imposed no limitations concerning the types of measures ([oxy-Hb] vs [deoxy-Hb]) or other specific details related to NIRS, such as the number of channels or sampling rate, for inclusion in this review.

### 2.2 Information sources

To identify potentially relevant records, we searched the PubMed database without any prespecified restriction. Records were also considered for inclusion if quoted in the reference lists of articles screened for inclusion. Our goal is to find out how VFT in fNIRS is differentially utilized in clinical settings between China and Japan. Thus, the final search strategy performed on PubMed is:

((VFT[Text Word] OR verbal fluency[Text Word]) AND (Chinese[Text Word] OR China[Text Word] OR Japanese[Text Word] OR Japan[Text Word])) AND (infrared[Text Word] OR nirs[Text Word]).

The search was in March 2023. Two reviewers independently charted the data and discussed the results; any disagreement was resolved by consensus with the involvement of a third team member.

### 2.3 Data processing

A preliminary data extraction form was designed by Liu. Two reviewers (Ren and Liu) independently charted the data and discussed the results; any disagreements were resolved by consensus, with the involvement of a third team member if necessary.

### 2.4 Data items

The data extraction process included several variables for each article, as follows:

(1)General information: The name of the author and year of publication.
(2)Sample characteristics: This involved demographic information, numerosity, and clinical data, including age, gender, clinical diagnosis, medication status, and any comorbid conditions present in the study population.
(3)NIRS specifics: This variable included information related to the positioning of probes, the number of channels, and the sampling rate used during data collection.
(4)Prefrontal activation tasks characteristics: This variable encompassed details regarding the type of prefrontal activation task employed in the study, such as verbal fluency, working memory, or emotional regulation.
(5)VFT specifics: This variable included information pertained to the utilizations of VFT in its clinical setting.
(6) Changes in hemodynamic parameters: This variable pertained to changes in hemodynamic parameters such as [oxy-Hb] and [deoxy-Hb] in the prefrontal cortex of patients.
(7) Potential findings regarding the relationship between NIRS measures and behavioral and clinical data: This variable included any potential findings related to the relationship between NIRS measures and behavioral and clinical data, such as correlations between hemodynamic parameters and symptoms severity, or medication response.

The findings of the studies selected were summarized in **Table 1** and some particular chosen to be elaborately analyzed in the main text.

**Table 1:** Summary of selected studies.

STUDY	SAMPLE N		AGE, mean ± SD			PATIENTS		NIRS SPECIFICS		VFT SPECIFICS		OXY-HB CHANGES		OTHER FINDINGS
Li et al. (34)	HC: 29 SZ: 47		HC: 29.2 ± 4.87 SZ: 29.04 ± 7.32			Chinese SZ		Number of chs: 52 (ETG-4100, Hitachi Medical Corporation, Tokyo, Japan) Sampling rate: 10 HZ Modes of fNIRS: CW Wavelength: 695 and 830 nm Probe’s position: frontal and superior temporal cortices (International 10–20 system).		Type: category/semantic (CFT,30s), Letter/phonemic (generate words with a given Chinese character, LFT, 30s) Control task: count 1-5 repeatedly (30s) Block composition: 1 control task (30s), then 1 activation task (semantic VFT), Repetitions: 4 blocks, then control task (post-task, 70s) (310s), then the same procedure for phonetic VFT; Modality: visual stimuli Performance: SP < HC in both CFT [(26.62 ± 6.53) vs. (41.17 ± 7.42)] and LFT [(17.00 ± 6.84) vs. (28.93 ± 8.65)]		HC: significant activation of 44 channels (all except Ch.2, 20, 32, 33, 36, 37, 43, and 47) in CFT, and 7 channels (Ch.7, 8, 18, 28, 29, 39, and 50) in LFT; SZ: significant activation of 33 channels (Ch.2-4, 6, 8, 13, 14, 17-19, 23-25,27-29, 34-41, and 44-52 during CFT and six channels (Ch.28, 29, 39, 40, 50, and 51) during the LFT		Left lateralization during CFT was reduced among schizophrenia patients and may be related to the semantic deficit. The Chinese- CFT could be a more sensitive indicator of frontal-temporal dysfunction in schizophrenia.
Quan et al. (47)	HC: 100 SZ: 165		HC: 34.43 ± 12.36 SZ: 33.81 ± 11.52			Chinese SZ		Number of chs: 52 (ETG-4000, Hitachi Medical Co., Japan) Sampling rate: 10 HZ Modes of fNIRS: CW Wavelength: 695 and 830 nm Probe’s position: frontal and superior temporal cortices (International 10–20 system).		Type: letter/phonemic (generate words with a given Chinese character, LFT, 30s) Task composition: pre-task baseline period (30s), activation VFT task (60s) and a post-task baseline (30s). Each character was presented for 2s and then replaced by the marker ‘+’ which lasted for 18s (a total of 20s for each cue character) Repetitions: No Performance: SP < HC in VFT [9.05 ± 3.90) vs. (11.29 ± 4.50)]. Modality: Visual stimuli		SZ<HC: schizophrenia patients had significantly lower increases of [oxy-Hb] than the healthy controls at 41 channels (Ch. 1-5, 9-16, 18-25, 27, 29-35, 39-46, and 49-52), lower decreases of [deoxy-Hb] at 24 channels (Ch. 12-13, 17, 22-24, 28-29, 32-35, 37-40, 43-46, and 48-51) and lower increases of [total-Hb] at 24 channels (Ch. 2-3, 11-13, 16, 19-21, 24-25, 30-31, 34-35, 39-42, 44-45, and 50-52).		Most channels showed negative correlations between age and [oxy-Hb] and the positive correlations between age and [deoxy-Hb] changes
Takizawa et al. (30)	HC: 70 SZ: 55		HC: 37.4 ± 13.6 SZ: 40.1 ± 11.1			Japanese SZ		Number of chs: 52 (ETG-4000, Hitachi Medical Co.) Sampling rate: 10 HZ Modes of fNIRS: CW Wavelength: 695 and 830 nm Probe’s position: bilateral prefrontal (approximately dorsolateral [Brodmann’s area (BA) 9, 46], ventrolateral [BA 44, 45], and frontopolar [BA 10]) and superior temporal cortical surface regions (International 10–20 system).		Type: letter VFT Control task: repeat Japanese vowels (/a/,/i/,/u/,/e/and/o/) aloud in pre-task and post-task Task composition: pre-task (30s), activation VFT (60s, 20s for each syllable), and post-task period (70s) Modality: Auditory stimuli		SZ<HC: Schizophrenia patients had significantly lower [oxy-Hb] increase than healthy subjects at 20 channels (Ch.17-18, 24-25, 28-29, 35-40, 42, 46-52)		Reduced frontopolar cortical activation is associated with functional impairment in patients with schizophrenia
Satomura et al. (48)	MDD: 45		MDD: 39.8 ± 11.8; Initial test and 1.5-year follow-up test			Japanese patients with major depressive disorder (MDD)		Number of chs: 52 (ETG-4000, Hitachi Medical Co., Japan) Sampling rate: 10 HZ Modes of fNIRS: CW Wavelength: 695 and 830 nm Probe’s position: bilateral prefrontal and temporal regions (International 10–20 system).		Type: letter VFT Control task: repeat Japanese vowels (/a/,/i/,/u/,/e/and/o/) aloud in pre-task and post-task Task composition: pre-task (30s), activation VFT (60s, 20s for each syllable), and post-task period (55s) Modality: NM		A significant negative correlation between Δ[oxy−Hb] and ΔHAMD was observed in Ch23.		Brain activation in the right IFG and the bilateral MFG as measured by NIRS may indicate clinical severity and trait-related abnormalities in MDD.
Takizawa et al. (10)	MDD:153; BP: 134; SZ: 136; HC: 590;		MDD:43.8 ± 12.7; BP: 44 ± 14.9 SZ: 43.7 ± 12.1 HC: 43.9 ± 15.7			Japanese patients with MDD, BP, and SZ.		Number of chs: 52 (ETG-4000; Hitachi Medical Co., Tokyo, Japan) Sampling rate: 10HZ Modes of fNIRS: CW Wavelength: 695 and 830 nm Probe’s position: bilateral prefrontal cortical area (i.e., dorso-lateral [Brodmann areas (BAs) 9 and 46], ventro-lateral [BAs 44, 45, and 47] and fronto-polar [BA 10] regions) and in the superior and middle temporal cortical surface region (International 10–20 system).		Type: letter VFT Control task: repeat Japanese vowels (/a/,/i/,/u/,/e/and/o/) in pre-task and post-task Task composition: pre-task (10s), activation VFT (60s, 20s for each syllable), and post-task period (70s) Modality: NM		Patients with MDD and those with BP/SZ revealed a significant difference in the R1 centroid values, but not in the R1 or the R2 integral value		Potential confounding effects of clinical (e.g., age, sex) and systemic (e.g., autonomic nervous system indices) variables on brain signals will need to be clarified to improve classification accuracy.
Pu et al. (49)	SZ: 41		SZ: 33.6 ± 11.2			Japanese SZ		Number of chs: 52 (ETG-4000; Hitachi Medical Co.) Sampling rate: 10HZ Modes of fNIRS: CW Wavelength: 695 and 830 nm Probe’s position: fronto-temporal region		Type: letter VFT Control task: repeat Japanese vowels aloud in pre-task and post-task Task composition: pre-task (30s), activation VFT (60s, 20s for each syllable), and post-task period (70s) Modality: NM		The mean [oxy-Hb] levels during the task were significantly higher than the pre-task baseline for 37 channels (Ch.2,9, 12, 13, 15, 19-26, 28-30,32-52) in SZ. The mean [deoxy-Hb] levels during the task period were significantly higher than those for the pre-task baseline for 28 channels (Ch.20, 23-25, 28-36, 38-52) in SZ.		Ventrolateral PFC, right dorsolateral PFC, and left temporal hemodynamic responses during the VFT were correlated with depression/anxiety component scores of the PANSS, even after controlling for VFT performance and demographic factors.
Nishimura et al. (50)	SZ: 73 HC: 73		HC: GG 38.9 , GA 37.6, AA 41.4 , SZ: GG 38.3, GA 36.1, AA 38.3			Japanese SZ		Number of chs: 52 (ETG-4000, Hitachi Medical Corporation, Tokyo, Japan) Sampling rate: 10HZ Modes of fNIRS: CW Wavelengths: 695 and 830nm Probe’s position: from the bilateral prefrontal cortical areas (e.g., frontopolar [FP; Brodmann’s area (BA) 10], dorsolateral [DL; BA 9, 46], and ventrolateral [VL; BA 44, 45, 47]) and superior temporal cortical surface regions (International 10–20 system).		Type: letter VFT Control task: repeat Japanese vowels aloud in pre-task and post-task Task composition: pre-task (10s), activation VFT (60s, 20s for each syllable), and post-task period (70s) Modality: NM		Patients with SZ exhibited a significantly smaller [oxy-Hb] increase during the VFT in 31 channels (Ch.1, 10, 11, 13, 14, 21, 24–29, 32, 34–40, 42–52; F = 5.8–23.7, FDR corrected p < 0.05)		Smaller DLPFC activation in EGR3-AA in healthy adults and in schizophrenia. The genetic variation in EGR3 may impact on PFC through neurodevelopment.
Ji et al. (51)	SZ: 200 HC:100		SZ: 33.81 ± 11.52			Chinese SZ		Number of chs: 52 (ETG-4000, Hitachi Medical Co., Japan) Sampling rate: 10HZ Modes of fNIRS: CW Wavelengths: NM Probe’s position: bilateral prefrontal and temporal regions (International 10–20 system).		Type: letter/phonemic (generate words with a given Chinese character, LFT, 30s) Task composition: pre-task baseline (30s, repeat 1-5 constantly), activation VFT task (60s) and a post-task baseline (30s) Repetitions: No Modality: Visual stimuli Performance: NM		Eleven channels (Ch.35,37,39,41,43-50,52) demonstrated significant between-group differences (t < - 2.82, p < 0.035, corrected by FDR).		Classification performance outperforms most of the results of the available studies.
Kiriyama et al. (52)	MDD:18 HC: 22		MDD:44.2 ± 8.9 HC: 42.0 ± 11.2			Japanese MDD		Number of chs: 52 (ETG-4000 Optical Topography System; Hitachi Medical Co., Tokyo, Japan) Sampling rate: 10HZ Modes of fNIRS: CW Wavelengths: 695 and 830nm Probe’s position: bilateral frontal, temporal, and parietal cortices according to LONI Probabilistic Brain Atlas (LPBA40)		Type: letter VFT Control task: repeat Japanese vowels aloud in pre-task and post-task Task composition: pre-task (30s), activation VFT (60s, 20s for each syllable), and post-task period (70s) Modality: NM		Mean VFT induced [oxy-Hb] changes were significantly lower in patients with MDD than in healthy individuals for 15 channels located in the bilateral prefrontal and temporal surface regions (Ch15, 22, 26-27, 30, 32-33, 38, 40-42, 44, 48, 51-52).		We suggest that reduced activation in the left temporal region in patients with MDD could be a biomarker of poor motor speed.
Wei et al. (53)	SZ:198 MD:54 BP:64 HC:101		SZ:38.19 ± 12.66 MD:34.06 ± 12.7 BP:34 ± 12.66 HC:27.55 ± 6.35			Chinese SZ, MDD, BP		Number of chs: 52 (ETG-4000, Hitachi Medical Co., Japan) Sampling rate: 10HZ Modes of fNIRS: CW Wavelengths: 695 and 830nm Probe’s position: bilateral prefrontal cortex as well as the bilateral temporal cortical areas (International 10-20 system)		Type: letter/phonemic (generate words with a given Chinese character, LFT, 30s) Task composition: pre-task baseline (30s, repeat 1-5 constantly), activation VFT task (60s) and a post-task baseline (70s) Repetitions: No Modality: Auditory stimuli		Between the collective patient group and HCs, significant differences in the IV of R1 and IV of R2 were found, but there were no significant differences for the CV of R1 and CV of R2. One-way ANOVA was performed between HC and patients with BP&SCH revealed a significant difference in the IV of R1 and R2, but not in the CV of either R1 or R2. BP&MD VS HC: Both R1 Integral and R2 Centroid value were included as statistically significant variables. SCH VS HC: This result was not very ideal.		We try to make a discrimination among those psychotic disorders; neither IV nor CV showed any significant difference.
Mesai et al. (54)	MDD:21		MDD:59.4 ± 12.0			Japanese MDD		Number of chs: 52 (ETG-4000; Hitachi Medical, Tokyo, Japan) Sampling rate: 10HZ Modes of fNIRS: CW Wavelengths: 695 and 830nm Probe’s position: bilateral prefrontal and superior temporal cortical surface regions (International 10-20 system)		Type: letter VFT Control task: repeat Japanese vowels (/a/,/i/,/u/,/e/and/o/) in pre-task and post-task Task composition: pre-task (30s), activation VFT (60s, 20s for each syllable), and post-task period (70s) Modality: NM		The mean VFT-related [oxy-Hb] changes averaged across 11 channels in the prefrontal region was 0.03, s = 0.06 (in mM·mm) in the remitted MDD patients group (n = 21). Those values averaged across 10 channels each in the left and right temporal regions were 0.07, s = 0.10 and 0.08, s = 0.10, respectively.		Remitted patients with MDD possibly have residual symptoms which are most likely to impair their social functioning and that these symptoms are differentially associated with brain function measured with NIRS.
Wang et al. (55)	first-episode MDD:36 recurrent MDD:34 HC:37		First-episode MDD:38.75 ± 13.86 recurrent MDD: 43.26 ± 13.85 HC: 35.7 ± 11.39			Chinese first-episode MDD and recurrent MDD		Number of chs: 52 (ETG-4000, Hitachi Medical Co., Japan ) Sampling rate: 10 HZ Modes of fNIRS: CW Wavelengths: 695 and 830nm Probe’s position: bilateral frontal and temporal cortices (International 10–20 system).		Type: letter/phonemic (generate words with a given Chinese character, LFT, 30s) Task composition: pre-task baseline period (30s), activation VFT task (60s) and a post-task baseline (30s). Each character was presented for 2s and then replaced by the marker ‘+’ which lasted for 18s (a total of 20s for each cue character) Repetitions: No Modality: Visual stimuli		The results of *post-hoc* tests revealed that the recurrent MDD group had significantly lower increases of oxy-Hb than the fMDD group in 4 channels (Ch.3 (Dorsolateral prefrontal cortex), 13 (pars triangularis Broca’s area), 33 (Superior Temporal Gyrus) & 34 (Inferior prefrontal gyrus). After Bonferroni correction, only 2 channels (Ch. 3 (Dorsolateral prefrontal cortex) & 13 (pars triangularis Broca’s area) emerged as significant.		Recurrent MDD had lower activation on right prefrontal cortex than first-episode MDD.

SZ, Schizophrenia; MDD, Major Depressive Disorder; BP, Bipolar Disorder; HC, Healthy Control; CFT, Category fluency task; LFT, Letter fluency task; IFG, inferior frontal gyrus; MFG, middle frontal gyri; R1, Region of interest 1; R2, Region of interest 2; EGR3-AA, AA genotype group of early growth response 3 (EGR3); IV, integral value; CV, centroid value; fMDD, first-episode MDD.

## 3 Results

### 3.1 Selection of sources of evidence

**Figure 1:**
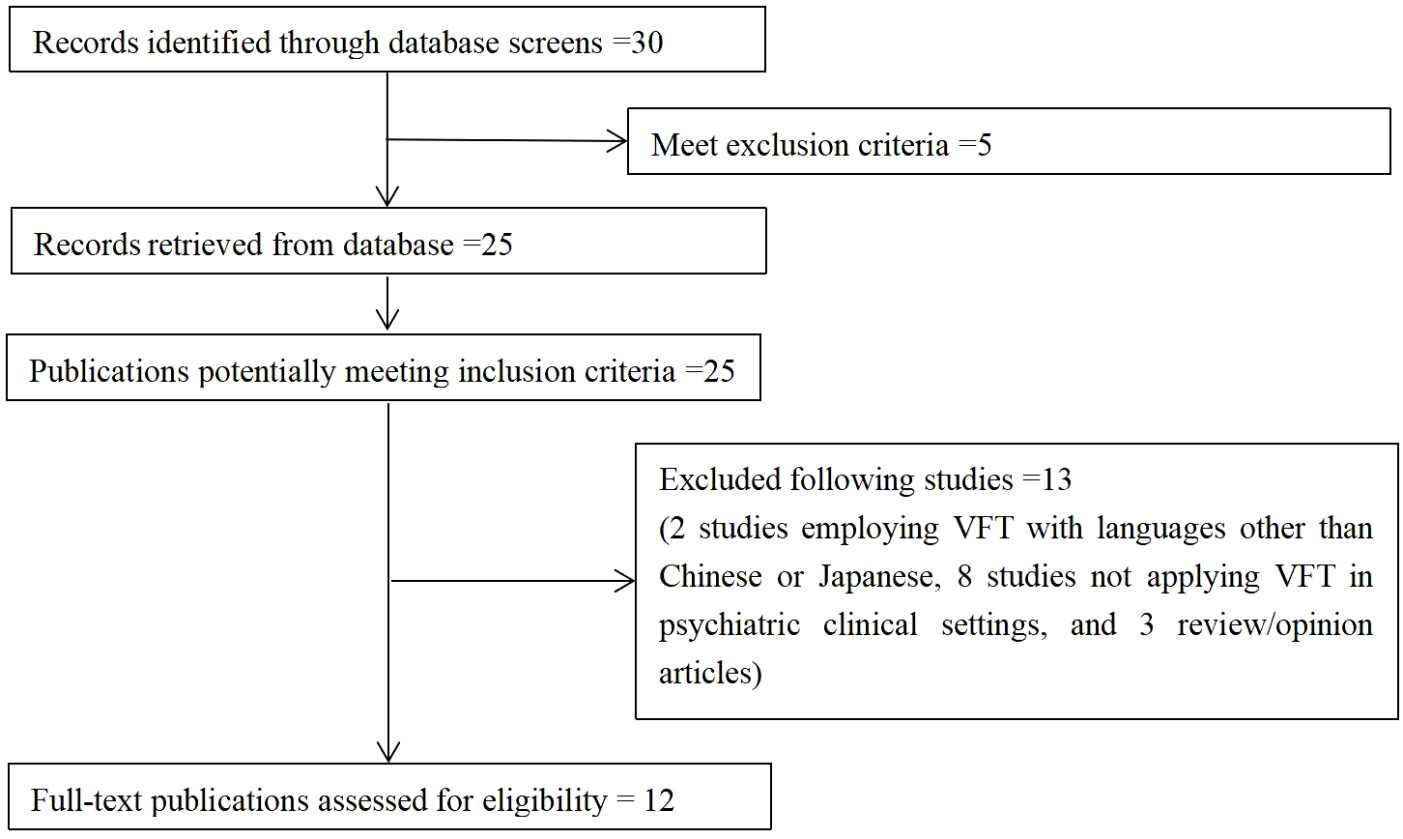
Flowchart of the studies selection process.

A total of 30 studies were retrieved from the literature and screened for eligibility (**Figure 1**). Five studies were excluded based on their title and abstract. We then reviewed the full text of the remaining 25 studies to assess their potential relevance for inclusion. Ultimately, 12 studies were included in the review. The 13 excluded studies were 2 studies employing VFT with languages other than Chinese or English, 8 studies not applying VFT in clinical settings, and 3 review/opinion articles. The characteristics of these included studies are summarized in **Table 1**.

### 3.2 Synthesis of results

fNIRS, in conjunction with VFT, is increasingly utilized in psychiatric diagnostics within clinical settings in China and Japan. Research articles examining VFT applications in these regions reveal divergent methodologies in terms of VFT task paradigm and its analysis methodology.

#### 3.2.1 VFT paradigms

VFT is a commonly employed neuropsychological test (1). It involves participants generating as many words as they can within a set time frame, either beginning with a specified letter or related to a certain semantic category (2). This has led to the development of two primary VFT variants: the letter VFT and the category VFT. In letter VFT, participants are prompted to produce words starting with a specific letter or sound, typically F, A, or S. In contrast, category VFT asks participants to generate words belonging to a pre-defined category, such as animals or fruit, as detailed by (56–58).

The VFT paradigm applied in Japan is fairly uniform, predominantly based on the letter VFT (10, 30, 48–50, 52, 54). In these studies, participants were instructed to generate as many Japanese words beginning with a designated syllable as possible during the activation period. Through the investigation of frontotemporal hemodynamics via NIRS, studies in Japan have explored the relationship between major depressive disorder (MDD) and schizophrenia (SZ) (28), MDD and bipolar disorder (BP) (29), functional impairment in SZ (30), and potential SZ and patients with first-episode psychosis (31).

Conversely, in China, two primary paradigms are utilized for assisting clinical diagnosis. One is an adapted version of the Japanese letter VFT, wherein Chinese characters such as “mountain”, “big”, “white”, and “sky” are given, and patients are required to produce word phrases involving the specified character (47, 51, 53, 55). The other paradigm is a multi-cognitive task approach based on the category VFT (see 59). This “Multi-cognitive Tasks NIRS System”, the first localized task to assist diagnosis in psychiatry in China, was developed by the research group of Professor Pozi Liu from Tsinghua University. It has assisted in diagnosing psychiatric disorders such as bipolar disorder and unipolar depression (32), major depressive disorder and generalized anxiety disorder (33), and schizophrenia (34), and has been granted a national invention patent (Invention Patent No. ZL201610342176.4).

For the types of VFT, both letter/phonemic VFT and category/semantic VFT are included. Japanese studies primarily utilize a standardized letter VFT, focusing on syllable-based word generation while Chinese clinical practice incorporates both an adapted version of the Japanese letter VFT, involving specific Chinese characters, and a multi-cognitive task approach centered around the category VFT.

#### 3.2.2 Analysis methodology

The analysis methodologies between studies in China and Japan differ significantly. In Japan, Takizawa et al. (10) introduced the “integral value (IV)” and “centroid value (CV)” metrics in a pioneering case-control study using near-infrared spectroscopy (NIRS) for diagnosing psychiatric disorders in a clinical setting. Employing a 52-channel NIRS system (ETG-4000; Hitachi Medical Corporation, Tokyo, Japan), they recorded oxy-Hb and deoxy-Hb at two infrared wavelengths based on the modified Beer-Lambert law (60). These metrics, IV and CV, quantified hemodynamic responses and temporal changes during a 60-second activation verbal fluency task (VFT). The IV quantifies the magnitude of the hemodynamic response during the 60-second activation VFT, whereas the CV serves as an index of temporal changes throughout the task (10). Subsequent Japanese studies adopted this protocol, incorporating paired t-tests and other statistical methods to confirm task-related brain activities (30, 48–52, 54).

**Figure 2:**
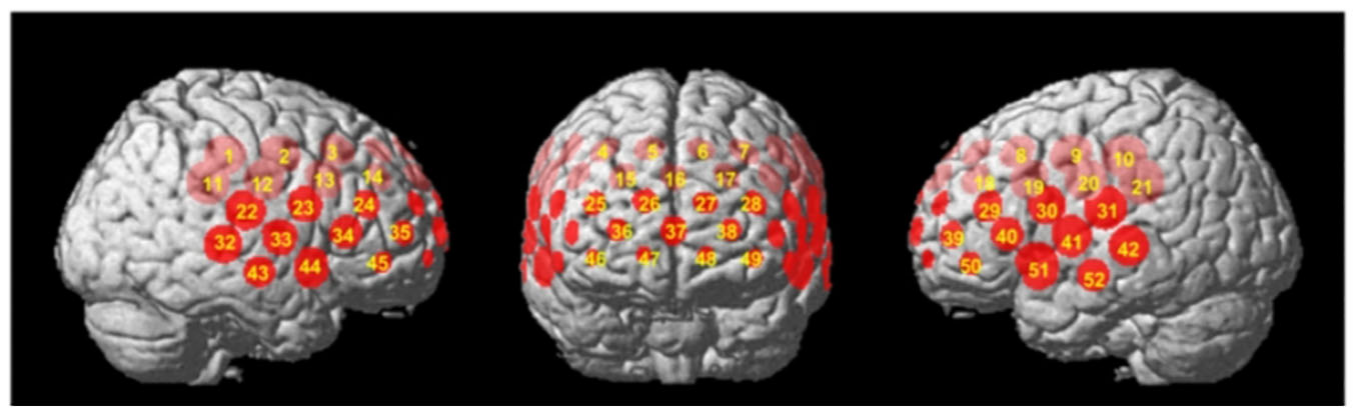
Regions of interest (Regions 1 and 2) of the near-infrared spectroscopy (NIRS) signals. The locations of near-infrared spectroscopy (NIRS) measurements were probabilistically estimated and anatomically labelled in the standard brain space (LBPA40) according to Tsuzuki et al. (62). Region 1: (ch 25–28, ch 36–38 and ch 46–49); Region 2, Right: (ch 22–24, ch 32–35 and ch 43–45); Left: (ch 29–31, ch 39–42 and ch 50–52) (cited from 10).

Initial analyses by Takizawa et al. (10) focused on individual and single-channel data. However, subsequent repeated NIRS measurements revealed that while NIRS signal reliability was acceptable at the group and cohort levels, it was unsatisfactory at individual and single-channel levels (61). As a result, Takizawa et al. employed principal component analysis (PCA) to examine changes in [oxy-Hb] signals at the individual level and in two cluster channels. The first cluster (R1) of NIRS signals consisted of channels located approximately in the fronto-polar and dorsolateral prefrontal cortical regions (i.e., superior and middle frontal gyri). Meanwhile, the second cluster (R2) of NIRS signals comprised signals from channels located approximately in the ventro-lateral prefrontal cortex and the superior and middle temporal cortical regions (i.e., inferior frontal gyrus and superior and middle temporal gyri) (see **Figure 2**, cited from 10).

This Japanese paradigm is a fitting analysis of selected channels, primarily focusing on language areas. Its waveform analysis mode involves a single-curve fit analysis of the relative concentration of [oxy-Hb] and area calculation of hyperbola at some selected channels, presented in a topographic map. This paradigm attempts to identify diagnostic features of the disease through waveform fitting (10).

In contrast, Chinese studies adopt a varied approach. Wei et al. (53) closely followed the Japanese methodology, using auditory tasks and ANOVA to analyze IV and CV in specific brain regions, revealing significant differences across psychiatric conditions but with limited specificity. This approach is distinct from the broader scope of Chinese research, which utilizes fNIRS to analyze full-channel oxy-Hb and deoxy-Hb concentrations across 45 channels. This method aims to discern differences in prefrontal working memory network regulation between normal and pathological states, linking waveform composition ratios with symptoms and disorders (59). Similar analytical methods are evident in other Chinese studies focusing on brain activation and its correlation with demographic and clinical variables (34, 47, 55).

Overall, the Japanese studies predominantly apply the methodologies of Takizawa et al. (10), emphasizing specific brain regions and tasks. In contrast, Chinese research encompasses a comprehensive approach, examining broader brain activation patterns to differentiate mental health states.

## 4 Discussion

To enhance the discussion on the neurolinguistic implications of the verbal fluency task (VFT) in clinical settings, particularly in China and Japan, it is essential to address the limitations and challenges inherent in these methodologies, including the variation in hemodynamic response patterns in fNIRS studies. Yeung and Lin (63) underscores the influence of linguistic factors on brain activation patterns in psychiatric disorders. The varied linguistic properties of cognitive tasks employed in different studies suggest a pivotal role for neurolinguistics in interpreting these variations. Such an analysis, considering phonetic versus semantic and visual versus auditory dimensions, highlights the importance of linguistic and cultural contexts in the neuropsychological assessment of psychiatric disorders.

### 4.1 Letter VFT and category VFT

Both letter/phonemic VFT and category/semantic VFT engage various aspects of lexical processing, including lexical retrieval and the activation of lexical memory from long-term storage, incorporating phonological, semantic, and syntactic features, as well as attention, abstract reasoning, and processing speed (64, 65). These tasks, while seemingly simple, engage complex psychological mechanisms and activate specific brain areas.

Neuroimaging studies further differentiate these tasks. Gourovitch et al. (66) and Jurado et al. (67) suggest that while some brain regions are commonly activated in both tasks (e.g., the anterior cingulate gyrus, left prefrontal area, thalamus, and cerebellum), others are distinct. The letter VFT more actively engages the inferior frontal and temporoparietal cortices, associated with phonological processing, whereas the category VFT more robustly activates the left temporal cortex, linked to semantic memory.

**Figure 3:**
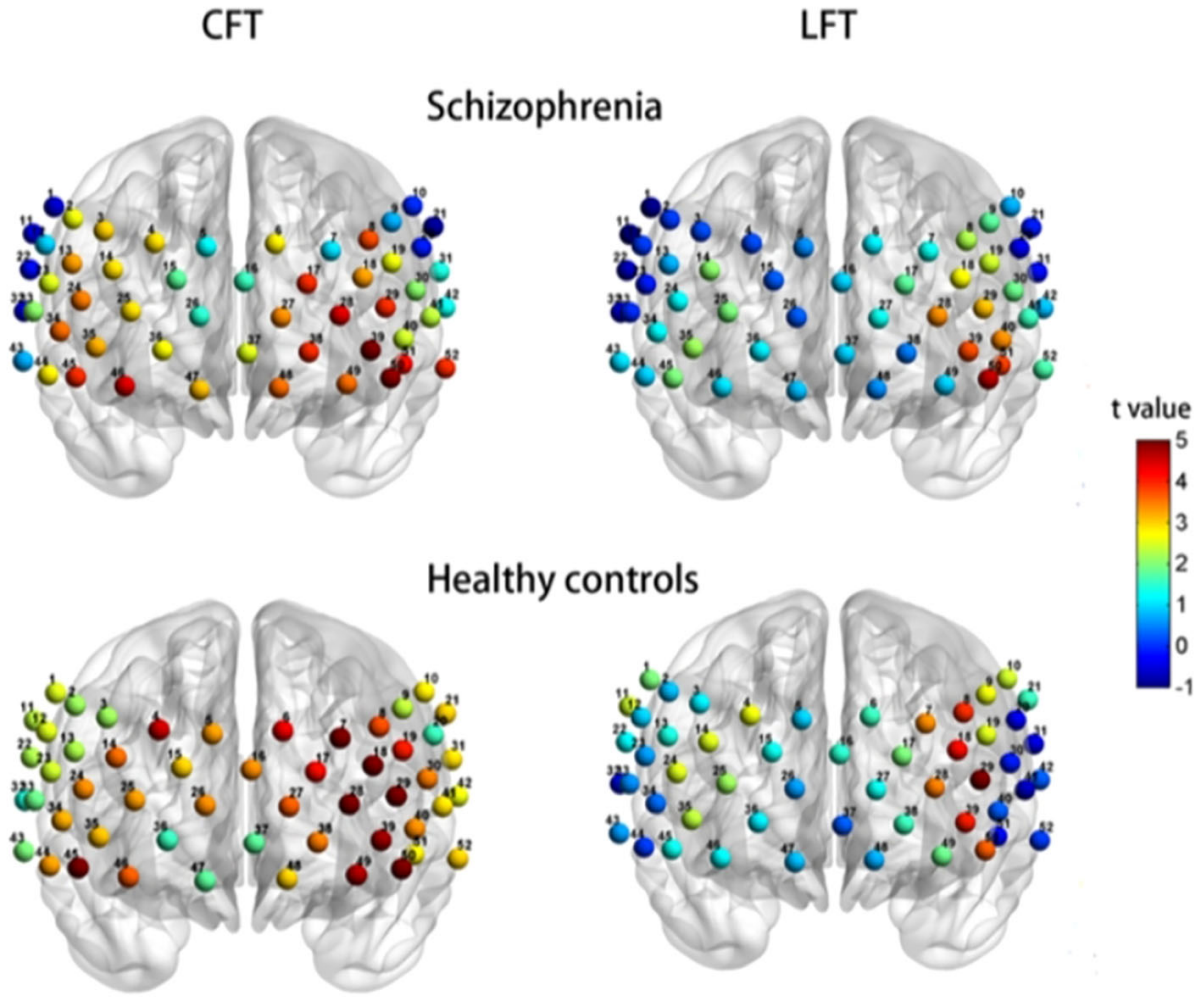
Activation of brain regions in the fNIRS Chinese category fluency task and letter VFT in schizophrenia versus healthy controls (cited from 34).

In Chinese clinical settings, a study by Li et al. (34) (see **Figure 3**) supports this double dissociation between letter and category VFT. Their research, focusing on Chinese schizophrenic patients and control subjects, demonstrates that the category VFT provides a clearer distinction in brain region activation, primarily engaging the left hemisphere’s language areas, while the letter VFT showed more limited activation. However, there is a noticeable gap in research comparing their neurolinguistic and neurophysiological implications, especially in terms of cerebral hemodynamics and oxygenation patterns. This calls for a more thorough investigation into how these VFT variants differentially affect brain function.

### 4.2 Modality differences

The impact of stimulus modality on cognitive processing and brain activation is significant (68–70). Auditory and visual stimuli engage different cognitive and neural pathways, influencing task performance. This variability underlines the need for more detailed studies on how these modalities affect cognitive performance and hemodynamic responses in fNIRS. Buchsbaum (70) elucidated the dual-pathway theory of auditory and visual stimulus inputs in the language processing mechanism. Research on brain activation and task processing reveals significant differences between auditory and visual modalities. Marinkovic et al.’s (68) study highlighted distinct brain activation patterns during oral and written language recognition, emphasizing the divergent pathways for auditory and visual processing. This finding aligns with the working memory model proposed by Baddley & Hitch (71), which separates the processing of visual and auditory stimuli, indicating that task performance can substantially vary depending on the modality.

The influence of modality on task performance is further exemplified by Rapp and Caramazza’s (72) study, which observed a stroke patient performing a synonym matching task. The results showed a higher accuracy rate (91%) with visual targets compared to auditory presentations (71%), suggesting that visual processing was more efficient in this context. The variability in processing efficiency between auditory and visual stimuli underlines the need for a nuanced understanding of these modalities, especially in contexts where optimizing cognitive performance is critical.

The specifics of the stimulus modality employed in the 2-minute and 5-second letter VFT in Takizawa et al.’s (10) research conducted in Japan were not stipulated. However, the adapted letter VFT in Chinese clinical settings presents notable deviations. While some studies employed visual stimuli (e.g., 47, 55), others utilized auditory stimuli (e.g., 53), and yet some did not specify the modality employed (73). Within the Chinese multi-cognitive tasks paradigm, the category VFT task requirements are presented in both visual and auditory forms. The difficulty in language processing varies across modalities and could potentially lead to differences in cognitive resource utilization, which in turn might affect hemoglobin consumption.

### 4.3 Language typology differences

The distinct language typologies of Japanese and Chinese influence VFT administration and processing (74–76). Understanding these typological differences is essential for accurately interpreting fNIRS data and their neurolinguistic implications. In languages with diverse orthographic and phonological structures, VFT activates different brain pathways (75). Thus, it is crucial to consider how language typological differences, including morphological, phonological, and syntactic variations, significantly influence the administration and processing of the VFT within various linguistic contexts.

The language typology of Japanese and Chinese is fundamentally disparate, leading to distinct neural mechanisms for language processing. Specifically, the Chinese language’s unique typological features, with its logographic system linking characters directly to meanings, play a critical role. This system, unlike alphabetic languages where graphemes map onto phonemes, results in the simultaneous activation of graphemes, phonemes, and semantics during character processing. Research indicates that while alphabetic languages typically activate the temporo-parietal and anterior temporal regions more robustly, Chinese characters predominantly stimulate areas like the middle frontal gyrus and inferior frontal gyrus (74, 76).

In contrast, Japanese language processing, particularly in mora-letter fluency tasks, presents another interesting case. In Japanese, a mora (the smallest phonological unit) corresponds to a “kana”, creating a tight mora-grapheme association. This differs from syllable-alphabet-based languages like English, where the first-letter search in a VFT is more flexible. In Japanese, the search is constrained to a specific mora, leading to the activation of additional brain areas not typically engaged in alphabetic languages. For instance, significant activations during the mora/letter fluency task are observed not only in the frontotemporal cortices, common to category fluency tasks, but also in the middle frontal gyrus and supramarginal gyrus in the left hemisphere, and overlapping with the angular gyrus in the right hemisphere (see 75). This increased neural activity could be related to the demands of speech processing, working memory, or the consumption of overall cognitive resources.

These examples highlight that language typology differences correspond to distinctive neural mechanisms, underscoring the need for a nuanced understanding of these variations, especially in the context of cognitive and linguistic assessments.

### 4.4 Limitations and challenges

Our goal is to synthesize research on hemodynamic responses induced by VFT in China and Japan, scrutinizing variations in task design (phonological versus semantic), stimulus modality (auditory versus visual), and language typology. Focusing on these countries is key, as it sheds light on the unique applications and adaptations of VFT in these linguistically and culturally distinct settings. By delving into these specific instances, our review highlights the need to customize VFT to suit various linguistic and cultural contexts, thus enhancing its validity in cross-cultural psychiatric evaluations. However, current discussions on VFT primarily concentrate on its neurolinguistic components and often overlook the comprehensive potential of fNIRS in cognitive tasks. This oversight leaves a notable gap in our understanding, as a thorough examination of hemodynamic responses across diverse studies is essential for a complete grasp of cognitive processes. Hemodynamic responses, which are changes in blood flow related to neural activity, can be intricately studied through fNIRS technology. Nevertheless, a systematic analysis of these response patterns, especially in the context of VFT, is scarcely addressed in the literature.

As for the challenges, there is a lack of a global perspective in VFT research, particularly in comparing findings from East Asian studies to those from other regions. East Asia presents unique linguistic and cultural contexts that might influence VFT responses, yet current research is limited in contrasting these findings with global data. This comparison is vital for understanding the neurolinguistics and neurophysiology aspects of VFT at a broader level.

Besides, exploring the impact of different diseases on Verbal Fluency Test (VFT) paradigms is essential for enhancing our understanding and diagnosis of mental health conditions. VFT paradigms, utilized to assess cognitive functions that require the orchestration of neural networks, particularly in the fronto-temporal regions, have shown significant promise in distinguishing between various psychiatric disorders.

Research leveraging Near-Infrared Spectroscopy (NIRS) to investigate fronto-temporal hemodynamics during letter VFT tasks has revealed critical insights. For instance, studies in Japan have elucidated the relationships between Major Depressive Disorder (MDD) and Schizophrenia (SZ) (28), MDD and Bipolar Disorder (BP) (29), as well as the functional impairments in SZ (30) and in individuals with first-episode psychosis (31). These findings suggest that VFT performance can reflect the unique neural disruptions associated with each condition. Similarly, in China, the category VFT has been instrumental in diagnosing psychiatric disorders such as bipolar disorder and unipolar depression (32), major depressive disorder and generalized anxiety disorder (33), and schizophrenia (34). These studies indicate that specific patterns of verbal fluency deficits may serve as markers for particular psychiatric conditions. However, the challenge of distinguishing between psychiatric diseases using VFT paradigms persists. This underscores the necessity for selecting the most appropriate VFT paradigm for diagnosis, tailored to the specific characteristics of the population being studied. Such a selection is crucial not only at a local level but also for making accurate cross-cultural comparisons. The effectiveness of VFT paradigms in diagnosing and differentiating mental diseases highlights the importance of further research in this area to refine our diagnostic tools and improve patient outcomes.

To enhance our comprehension, it is crucial to incorporate a comparative analysis of VFT findings at behavioral, cerebral, and physiological levels between East Asia and other global regions. This comparative approach would enable researchers to discern universal versus culture-specific patterns in VFT responses. Such differentiation is especially significant in the context of mental health diagnostics, where understanding these patterns can contribute to developing more accurate and culturally sensitive diagnostic tools. Given the cultural nuances in language processing and cognitive functioning, understanding these variations is not just an academic pursuit but also a practical necessity in the field of mental health. Culture-specific patterns in cognitive tasks like VFT can reveal important insights into how mental health conditions manifest and are experienced across different cultures.

## 5 Conclusion

The application of fNIRS in psychiatric diagnosis is still an emerging field, with significant potential yet to be fully realized. To effectively integrate fNIRS into clinical practice, extensive research is required, particularly involving diverse cognitive tasks and larger participant cohorts. Establishing robust baseline data through such research is vital for improving the accuracy of clinical diagnoses among patients with psychiatric conditions.

This research endeavor is a pioneering step in comparing the use of the fNIRS VFT paradigm in clinical psychiatry between China and Japan, framed within a neurolinguistic context. A critical aspect of this research is acknowledging the subtle differences in letter/category fluency tasks, stimulus modalities, and linguistic typologies. These factors are instrumental in developing a standardized VFT protocol that can be reliably used in clinical settings.

Developing such a protocol has far-reaching implications. It would not only deepen our understanding of psychiatric conditions in patients but also enable effective cross-cultural comparisons in clinical research. This is particularly important given the cultural and linguistic diversity across patient populations. By addressing these gaps in the current literature and practice, the goal is to enhance the diagnosis and treatment of psychiatric disorders, making it more tailored and effective for individuals from various cultural and linguistic backgrounds. This approach represents a significant stride towards a more inclusive and precise practice in psychiatric care, leveraging advanced neuroimaging technologies like fNIRS.

## Data availability statement

The original contributions presented in the study are included in the article/supplementary material. Further inquiries can be directed to the corresponding author.

## Author contributions

YR: Writing – original draft, Writing – review & editing. GC: Supervision, Writing – review & editing. KF: Writing – review & editing. XZ: Writing – review & editing. CY: Writing – review & editing. PL: Conceptualization, Supervision, Writing – review & editing.
